# Association between inflammation and skeletal muscle proteolysis, skeletal mass and strength in elderly heart failure patients and their prognostic implications

**DOI:** 10.1186/s12872-020-01514-0

**Published:** 2020-05-15

**Authors:** Masayuki Koshikawa, Masahide Harada, Shunsuke Noyama, Ken Kiyono, Yuji Motoike, Yoshihiro Nomura, Asuka Nishimura, Hideo Izawa, Eiichi Watanabe, Yukio Ozaki

**Affiliations:** 1 Department of Cardiology, Fujita Health University School of Medicine, 1-98 Dengakugakubo, Kutsukake-cho, Toyoake, Aichi 470-1192 Japan; 2 Division of Bioengineering, Graduate School of Engineering Science, Osaka University, Toyonaka, Japan; 3 Department of Cardiology, Fujita Health University School of Medicine, Bantane Hospital, Nagoya, Japan

**Keywords:** Elderly, Sarcopenia, Frailty, Mortality

## Abstract

**Background:**

Inflammation and skeletal muscle wasting often coexist in elderly populations, but few studies have examined their relationship in elderly heart failure (HF) patients. This study examined the relationship between inflammation and increased skeletal muscle proteolysis, reduced skeletal mass and strength, and their prognostic implications in elderly HF patients (> 65 years) using a random forest approach.

**Methods:**

We prospectively enrolled consecutive elderly HF patients (*n* = 78) and age- and sex-matched control subjects (*n* = 83). We measured the interleukin (IL)-6, C-reactive protein (CRP), and B-type natriuretic peptide (BNP) levels, lower limb muscle mass and strength, and 6-min walk distance. The amount of muscle proteolysis was determined by urinary 3-methylhystidine, normalized by creatinine (3-MH/Cr). The composite endpoint was defined as all-cause death or hospitalizations due to worsening HF.

**Results:**

Compared to controls, elderly HF patients had a significantly higher IL-6, CRP, BNP, and 3-MH/Cr, and exhibited a reduced lower limb muscle mass and strength. A correlation analysis demonstrated significant positive correlations between the inflammatory cytokine levels and 3-MH/Cr and BNP, and negative correlations with the lower limb muscle mass and strength, and 6-min walk distance. During a median follow-up of 2.4-years, 24 patients reached the endpoint. A random forest model revealed that inflammatory cytokines, skeletal muscle wasting, and the BNP had greater effects on the risk prediction. The algorithm achieved an area under the receiver operating characteristic curve of 0.887 (95% CI, 0.772–1.000).

**Conclusion:**

This study provided evidence of the association between inflammation and increased skeletal muscle proteolysis, reduced skeletal mass and strength, and their prognostic roles in elderly HF patients.

## Background

Age-related loss of the skeletal muscle mass and function, so called sarcopenia, can lead to physical disability and an increased risk of death [1, 2]. Although the etiology of sarcopenia remains multifaceted, age-related chronic inflammation has been implicated in muscle wasting and related sequelae. In early studies, higher levels of interleukin (IL)-6 and C-reactive protein (CRP) have been shown to be associated with a greater loss of muscle mass and strength in a community-dwelling elderly population [3, 4]. Recent evidence suggests that the signaling pathways related to inflammation may be associated with anabolic deficiencies and/or excessive muscle proteolysis [5].

Heart failure (HF) has become a significant global epidemic for patient populations of all ages; however, in the elderly, there may be differences in the co-morbidities and outcomes compared to younger HF patients [2, 6–8]. One co-morbidity that has received increasing attention in elderly HF is sarcopenia. Examination of muscle biopsies obtained from patients with HF demonstrated fiber atrophy and increased numbers of inflammatory cells [8]. These findings were associated with elevated levels of the inflammatory cytokines, suggesting a potentially shared pathogenesis of skeletal muscle atrophy in elderly HF and aged people and implicate systemic and/or local inflammation as a potential causative mechanism of skeletal muscle wasting.

Examination of the association between inflammatory cytokines and muscle proteolysis that promote muscle loss is important because it may yield targets for therapeutic intervention. Thus, the aim of this study was to examine whether increased inflammatory cytokines were correlated with excessive proteolysis and a greater loss of skeletal muscle mass, strength, and function in elderly HF patients. Furthermore, we investigated the prognostic role of the inflammatory cytokine levels and skeletal muscle wasting using a random forest approach, one of the machine learning techniques widely used in various scientific fields [9].

## Methods

### Patients

We prospectively enrolled consecutive elderly HF patients and age- and sex-matched control subjects in the cardiology department between January 2014 and June 2016. The eligibility requirements of HF at the time of screening included an age > 65 years, a B-type natriuretic peptide (BNP) level > 100 pg/mL and received an optimal stable pharmacological therapy [10]. In addition, they had a history of hospitalization due to HF but had no hospitalization due to HF in the 3 months prior to enrollment. The HF patients presented with symptoms (e.g. breathlessness, ankle swelling and fatigue) that were accompanied by signs (e.g. elevated jugular venous pressure, pulmonary crackles and peripheral edema) of worsening HF upon hospitalization, which improved with pharmacological treatment and/or mechanical circulatory support [11]. We did not consider the left ventricular ejection fraction (LVEF) as a criterion for enrollment. We excluded those patients who had acute infectious diseases, connective tissue diseases, chronic kidney disease (estimated glomerular filtration rate < 30 mL/min/1.73 m^2^), heart transplantations, had a systolic blood pressure > 180 mmHg, and/or a diastolic blood pressure > 100 mmHg. We also excluded any patients who had experienced myocardial infarctions, angina pectoris, or strokes/transient ischemic attacks within the last 6 months, lower limb disabilities, or orthopedic pain or neurologic symptoms.

### Assessment of skeletal muscle proteolysis

- We measured urinary 3-methylhistidine (3-MH) to assess skeletal muscle proteolysis. The amino acid, 3-MH, is generated by post-translational methylation of histidine residues derived from actin and myosin degradation [12]. It cannot be further catabolized, or recycled for protein synthesis, and is excreted in the urine. Therefore, it reflects the myofibril protein turnover [13]. All patients collected 24-h urine into bottles containing hydrochloric acid. The 3-MH in the urine was measured by a precolumn derivatization using reagent 3-aminopyridyl-*N*-hydroxysuccinimidyl carbamate (APDSTAG WAKO, FUJIFILM WAKO Inc. Osaka, Japan), followed by reversed-phase high-performance liquid chromatography and electrospray ionization tandem mass spectrometry (LCMS-2020, Shimadzu Corporation, Kyoto, Japan). The urine creatinine was analyzed by a Jaffe reaction. The amount of skeletal muscle proteolysis was determined as the ratio of the urinary 3-MH to creatinine (3-MH/Cr) because 3-MH is influenced by the renal function. All patients were asked to refrain from ingesting soy-based products and meat 3 days before the measurement because of a possible effect on the 3-MH.

### Assessment of lower limb muscle mass and strength

The lower limb skeletal muscle mass was assessed by the thigh circumference and thigh muscle thickness (thickness of the rectus femoris and vastus intermedius) of the dominant lower limb. The thigh circumference was measured 10 cm above the knee. The thigh muscle thickness was measured at the midpoint of the line connecting the upper border of the knee and anterior superior iliac spine using B-mode ultrasonography at 3-MHz, with a 4.6 cm linear transducer array (iE33, Philips, Amsterdam, The Netherlands) by a single sonographer unaware of clinical information [14]. The lower limb skeletal muscle strength was assessed by an isometric maximum voluntary contraction of knee extensor in the dominant leg using a portable dynamometer (μTas F-100, ANIMA, Tokyo, Japan) [15]. All subjects were studied while seated in a straight-backed chair. The pelvis was secured with a belt to prevent hip joint extension. The lower leg was kept dependent and the knee flexed to 90° with its posterior aspect positioned at the front edge of the chair. The force was measured with a strap looped around the dominant leg just above the ankle. The knee extensor strength was measured 3 times, and the greatest strength was used for the analysis. The knee extensor strength was expressed in kg.

### Six-minute walk test

A 6-min walk distance test was conducted to assess the physical performance. In this test, the patients completed as many laps of a 30 m walkway as they could in a 6 min period, supervised by a laboratory technician.

### Blood samples and echocardiography

- Blood tests and two-dimensional echocardiography were performed upon study enrolment. To avoid the effect of exercise testing on the IL-6, blood sampling was performed following echocardiography in the supine position. Blood samples were collected from a peripheral vein into tubes containing aprotinin and ethylene diamine tetra acetic acid. The blood samples were centrifuged at 1000×g at 4 °C for 15 min to isolate serum or plasma and store it at − 80 °C until assayed. The plasma BNP concentration was measured using a chemiluminescence enzyme immunoassay for human BNP (LLMIPULSE Presto, Fujirebio Inc. Tokyo, Japan). The serum IL-6 concentration was measured using a chemiluminescence enzyme immunoassay for human IL-6 (human IL-6 measurement kit, Fujirebio Inc. Tokyo Japan) and serum high-sensitivity C-reactive protein (CRP) levels via a latex-enhanced hs-CRP immunoassay (N-Latex CRP II, Siemens Healthineers, Tokyo, Japan). A single echocardiographer who was blinded to the patients’ clinical information performed offline echocardiographic analysis using a Vivid 7 (GE Healthcare, Boston, Massachusetts, USA).

### End point and follow-up

The end point was defined as either all-cause death or hospitalizations due to worsening HF. The follow-up data of the patients were determined from their medical records. The data of those who did not revisit our hospital were obtained by a telephone interview of the patient, patient’s family, or family doctor. The study protocol was approved by the ethics committee of the Fujita Health University School of Medicine, approval number 14–031. Each participant provided written informed consent. The investigation was performed in accordance with the ethical standards as laid down in the 1964 Declaration of Helsinki and its later amendments.

#### Statistical analysis

Continuous variables are presented as either the mean ± standard deviation (SD) value or median [interquartile range, IQR], and categorical variables as the frequency and percentage. Continuous variables were compared with Student’s t-test and χ^2^ test was used for categorical data comparisons. The relationship between the clinical variables was assessed by a Spearman’s rank correlation analysis. We used a random forest approach for survival analysis [9]. A random forest consists of many individual decision trees, where a decision tree is a flowchart-like diagram that shows a series of judgment conditions (if-then rules). Each individual tree in the random forest provides a class prediction (e.g., categorized as having a high or low mortality risk) independently, and the class with the most votes becomes the final prediction. The fundamental idea behind the random forest is the wisdom of crowds. While some tree decisions in the forest may be wrong, many other decisions would be right. Therefore, as a group, the forest’s decision can move in the correct direction. The random forest, like a logistic model classifier, provides a probability estimate (ranging from 0 to 1) for each patient to belong in the high mortality class. Using the value of the estimated probability, we drew the receiver operating characteristic (ROC) curve. An optimal cutoff value (cutoff probability) was defined as the point closest to the point (0, 1) on the ROC curve. We considered 29 variables for this analysis **(**Supplementary file 1). To evaluate the importance of each covariate, the mean decrease impurity (Gini importance) was measured on a training dataset. Only covariates of the mean decrease impurity > 0 were used for testing. The performance of the model was assessed by the leave-one-out cross-validation. Each random forest model was comprised of 1000 trees, which provided a robust classification in determining the prognosis for each individual patient. We also performed a multiple logistic regression analyses using backward elimination procedure. The time-to-event curve describing the proportion of patients remaining endpoint-free was calculated by the Kaplan-Meier method and compared with the log-rank test. A two-tailed *p*-value of < 0.05 was considered significant. Statistical analyses were performed using JMP 10.0.2 software (SAS Institute, Cary, NC, USA), the Scikit-learn machine learning library in Python and R-project for Statistical Computing 3.2.2 software.

## Results

### Clinical characteristics of the patients

The patient characteristics are listed in Table 1. The average age of the total 161 patients was 72 ± 7 years and 32% were female. Compared with the controls (*n* = 83), the HF patients (*n* = 78) had a higher CRP, IL-6 and BNP. Also, HF patients exhibited a lower hemoglobin, reduced LVEF compared to the controls. In the control subjects, 10 patients had an LVEF< 50%.

**Table 1:** Clinical characteristics of the patients

Characteristic	Control (n = 83)	Heart failure (n = 78)	P-value
Age (years)	72 ± 5	73 ± 8	0.21
Female	24 (29)	28 (36)	0.15
Body mass index (kg/m^2^)	23.3 ± 3.5	22.8 ± 4.2	0.42
Systolic blood pressure (mmHg)	123 ± 17	125 ± 23	0.69
Diastolic blood pressure (mmHg)	75 ± 13	76 ± 14	0.51
Cardiac rhythm			0.40
Sinus rhythm	35 (42)	23 (29)	
Paroxysmal/persistent AF	48 (58)	55 (71)	
Medical comorbidities			
Coronary artery disease	6 (7)	6 (8)	0.86
Valvular disease	1 (1)	10 (13)	< 0.001
Hypertension	30 (36)	38 (49)	0.11
Diabetes	10 (12)	16 (21)	0.14
Dyslipidemia	17 (20)	14 (18)	0.68
TIA/Stroke	4 (5)	11 (14)	< 0.05
Laboratory data			
Hemoglobin (g/dL)	13.8 ± 11.7	12.7 ± 2.0	< 0.001
Creatinine (mg/dL)	0.83 ± 0.19	0.88 ± 0.39	0.36
eGFR (mL/min/1.73m^2^)	68 ± 17	66 ± 18	0.39
CRP (mg/dL)	0.16 ± 0.26	0.70 ± 1.16	< 0.001
IL-6 (pg/mL)	3.30 ± 3.71	8.08 ± 7.05	< 0.001
Creatine kinase (IU/L)	91 ± 33	92 ± 50	0.87
BNP (pg/mL)	41 ± 28	328 ± 249	< 0.001
LVEF (%)	56 ± 11	45 ± 16	< 0.001
LAD (mm)	35 ± 5.8	40 ± 7.4	< 0.001
Medications			
β-Blocker	59 (71)	75 (96)	< 0.001
ACE-I/ARB	29 (35)	66 (85)	< 0.001
Loop diuretics	4 (5)	31 (40)	< 0.001
Aldosterone blocker	5 (6)	18 (23)	< 0.01
Calcium blocker	23 (28)	19 (24)	0.56
Statin	16 (19)	27 (35)	< 0.05
Aspirin	10 (12)	21 (27)	0.16
Anticoagulant	48 (58)	56 (72)	0.09

*AF* Atrial fibrillation, *TIA* Transient ischemic attack, *eGFR* Estimated glomerular filtration rate, *CRP* High-sensitivity C-reactive protein, *IL-6* Interleukin 6, *BNP* B-type natriuretic peptide, *LVEF* Left ventricular ejection fraction, *LAD* Left atrial dimension, *ACE-I* Angiotensin converting enzyme inhibitor, *ARB* Angiotensin II type 1 receptor blocker. Data represent the number, frequency, or means±SD

### Lower limb skeletal muscle measurements and the 6-min walk test

The results of the skeletal muscle measurements and 6-min walk test are summarized in Table 2. The HF patients had a smaller thigh circumference, reduced thigh muscle thickness, and a weaker knee extensor strength compared to the controls. The 3-MH/Cr in the HF patients was greater than that in the controls. The 6-min walk distance was significantly shorter in the HF patients.

**Table 2:** Skeletal muscle measurements and the 6-min walk distance

	Control (n = 83)	Heart failure (n = 78)	P-value
Thigh circumference (cm)	48 ± 7.2	42 ± 8.2	< 0.001
Thigh muscle thickness (cm)	2.9 ± 0.2	1.8 ± 0.3	< 0.001
Knee extensor strength (kg)	19.7 ± 6.4	13.9 ± 4.4	< 0.001
3-MH/Cr (nmol/g Cr)	191 ± 144	386 ± 445	< 0.001
6-min walk distance (m)	570 ± 45	534 ± 45	< 0.001

*3-MH* 3-methylhystidine, *Cr* Creatinine. Data represent means±SD

### Spearman’s rank correlation analyses

The relationships between the clinical features and skeletal muscle measurements in the control and HF patients are shown in the Tables 3 and 4. The IL-6 or CRP did not have significant correlations with thigh circumference, rectus femoris thickness, and 3-MH/Cr in the control subjects. In the HF patients, however, there were significant positive correlations between the inflammatory cytokines (IL-6 and CRP) and 3-MH/Cr and BNP, and negative correlations with the rectus femoris thickness, knee extensor strength, and 6-min walk distance.

**Table 3:** Relationships between the clinical features and skeletal muscle measurements in the controls

	Age	Female	AF	CRP	IL-6	BNP	LAD	LVEF	Thigh circumference	Rectus femoris thickness	Knee extensor strength	3-MH/Cr
Female	− 0.03											
AF	−0.14	− 0.04										
CRP	−0.06	− 0.16	− 0.12									
IL-6	0.08	−0.13	− 0.18	0.53^§^								
BNP	0.02	0.21	0.13	0.07	0.17							
LAD	−0.05	− 0.10	0.16	0.26^*^	0.27^*^	0.43^§^						
LVEF	−0.03	0.05	0.01	−0.22^*^	− 0.26^*^	− 0.15	− 0.19					
Thigh circumference	−0.29^*^	− 0.40^#^	− 0.15	−0.15	− 0.03	− 0.40^#^	− 0.17	−0.02				
Rectus femoris thickness	−0.32^*^	−0.40^#^	− 0.11	−0.12	− 0.10	−0.16	− 0.02	0.09	0.58^§^			
Knee extensor strength	−0.37^#^	−0.32^#^	− 0.10	−0.10	− 0.15	−0.31	− 0.04	0.16	0.43^#^	0.64 ^§^		
3-MH/Cr	0.22^*^	0.16	−0.17	0.09	0.13	0.17	0.03	−0.16	0.05	−0.14	−0.23^*^	
6-min walk distance	−0.29^*^	−0.14	− 0.07	−0.30^#^	− 0.57^§^	−0.41^#^	− 0.43^§^	0.18	0.34^#^	0.29^*^	0.25^*^	−0.22^*^

The abbreviations are as in Tables 1 and 2. ^*^P < 0.05, ^#^*p* < 0.01, ^§^*p* < 0.001

**Table 4:** Relationships between the clinical features and skeletal muscle measurements in the heart failure patients

	Age	Female	AF	CRP	IL-6	BNP	LAD	LVEF	Thigh circumference	Rectus femoris thickness	Knee extensor strength	3-MH/Cr
Female	−0.05											
AF	−0.13	0.13										
CRP	0.13	0.10	0.11									
IL-6	0.22	0.07	0.21	0.51^§^								
BNP	0.31^#^	0.04	0.31^#^	0.31^#^	0.50^§^							
LAD	0.05	−0.07	0.22^*^	0.16	0.05	0.24^*^						
LVEF	0.08	−0.08	− 0.23	−0.32^§^	− 0.52^§^	−0.49^§^	− 0.20					
Thigh circumference	−0.41^#^	−0.14	0.13	−0.17	− 0.22	−0.40^#^	− 0.02	0.32^#^				
Rectus femoris thickness	−0.35^#^	−0.31^*^	0.07	−0.27^#^	− 0.41^§^	−0.42^§^	− 0.05	0.31^#^	0.49^§^			
Knee extensor strength	−0.36^#^	−0.21	0.05	−0.36^#^	− 0.41^§^	−0.60^§^	− 0.14	0.43^§^	0.50^§^	0.69^§^		
3-MH/Cr	0.09	0.17	0.26^*^	0.21^#^	0.28^#^	0.26^#^	0.14	−0.12	−0.26^*^	−0.24^*^	− 0.22^*^	
6-min walk distance	−0.19	−0.14	0.10	−0.42^§^	− 0.77^§^	−0.42^§^	− 0.16	0.46^§^	0.23^*^	0.32^*^	0.37^#^	−0.28^*^

The abbreviations are as in Tables 1 and 2. ^*^P < 0.05, ^#^*p* < 0.01, ^§^*p* < 0.001

### Random forest and survival analyses

**Fig. 1:**
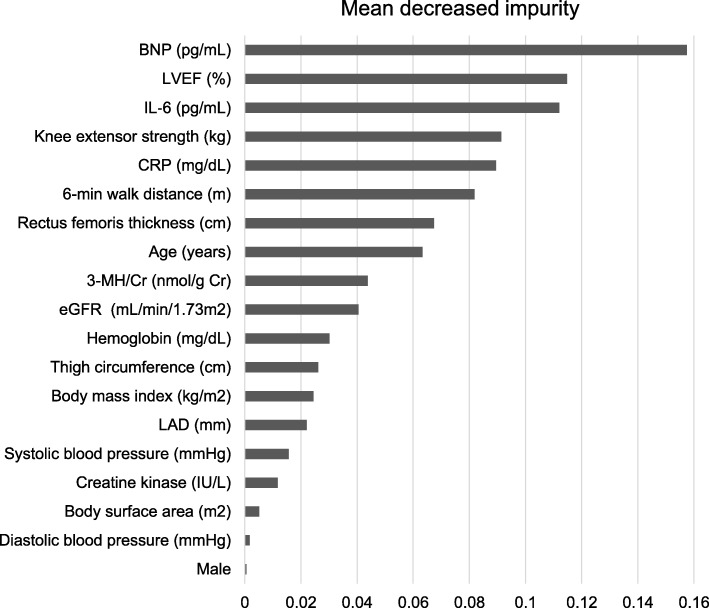
The mean decreased impurity from cross-validation procedure. This figure is sorted by the order of contribution of each variable for risk prediction. The random forest model selected 19 variables

**Fig. 2:**
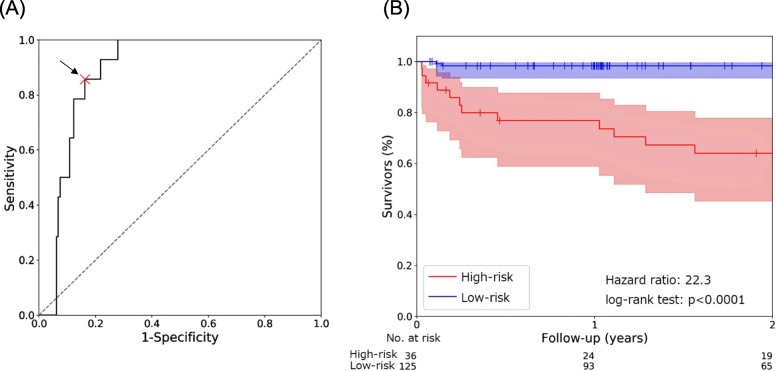
**a** Receiver operating characteristics curve. The cutoff points for the dichotomization (0.499) were determined by maximizing the sensitivity and specificity of the receiver-operating characteristics curve. **b** Kaplan-Meier survival analysis. The Kaplan-Meier analysis demonstrated a significantly higher event rate in patients classified as a high-risk group by a random forest model (hazard ratio 22.3, log-rank test, *p* < 0.0001)

During the median follow-up period of 2.4 years [1.0–3.6 years], 24 patients reached the endpoint. In the control subjects, no patients died but 2 hospitalized due to HF. In the HF patients, 15 died of HF (*n* = 7) or sudden/unwitnessed death (*n* = 8), and 7 hospitalized due to HF. Figure 1 shows the results of mean decrease impurity of the leave-one-out cross-validation. The BNP followed by LVEF, inflammatory cytokines, and knee extensor strength were the highest ranked feature for a risk prediction. Four of the top 10 ranked features were skeletal muscle-related parameters (knee extensor strength, 6-min walk distance, rectus femoris thickness and 3-MH/Cr). A female sex did not contribute to the risk prediction. Using a ROC curve (Fig. 2a), we calculated the optimal cut-off value to identify the patients in the high-risk group and low-risk group. This had a sensitivity of 83.7% and specificity of 85.7%. The area under the ROC curve was 0.887 (95% confidence interval 0.772–1.000). The Kaplan-Meier plots are shown in Fig. 2b. A multiple logistic regression analyses revealed that the area under the ROC curve was 0.674 (95% confidence interval 0.514–0.835), which was significantly lower (*p* < 0.01) than that in the random forest approach (Supplementary File 2). It showed that the BNP, age and systolic blood pressure were independent predictors.

## Discussion

In this study we investigated the associations between the inflammatory cytokines and skeletal muscle proteolysis, muscle mass, and strength in elderly HF patients. Furthermore, we examined their prognostic role using a random forest approach. We found that [1] elderly HF patients had excessive muscle proteolysis, and reduced skeletal muscle mass and strength compared to controls, [2] inflammatory cytokines and BNP were positively associated with increased muscle proteolysis and negatively associated with muscle mass and strength, and [3] the random forest approach could be effectively applied to predict outcome.

Skeletal muscle is a major reservoir of proteins in the body and is involved in its endocrine and metabolic activity as well as immune function [1, 2, 6]. A reduced skeletal muscle mass and strength are significant co-morbidities in elderly HF patients and lead to weakness, loss of independence, and an increased risk of death [1, 7]. A previous study has shown a higher prevalence of muscle wasting in elderly HF patients compared with healthy subjects of the same age [16]. The finding of this research is consistent with our study, where elderly HF patients had lower muscle mass and strength compared to age- and sex-matched controls. We further found that elderly HF patients had a greater loss of skeletal muscle evidenced by an increased excretion of urinary 3-MH/Cr despite relatively preserved 6-min walk distance. This observation suggests that even small imbalances of muscle synthesis and proteolysis could eventually lead to reduced skeletal muscle mass and strength.

The contractile proteins of skeletal muscle including actin and myosin, are methylated following peptide bond synthesis, with production of 3-MH. The measurement of the 3-MH excretion has been shown to provide a reliable measure of muscle protein turnover because the 3-MH is not reused for protein synthesis and has an excretion half-life of 12.2 h [12, 13, 17–22]. Studies on the quantitative excretion following (^14^C) 3-MH administration in humans have shown that no (^14^C) 3-MH is expired in the breath, while 99% of (^14^C) 3-MH is detected in the urine in 48 h. Furthermore, the urinary radioactivity rose progressively from 40% in rats weighing 50 g to 90% in animals weighing over 250 g [17]. This result suggested that proteolysis increased in proportion to the increase in the muscle mass. In the clinical setting, previous studies examined the turnover rate of muscle proteins using 3-MH/Cr under various nutritional conditions including high and low dietary protein levels [23], starvation [24], and glucocorticoid administration [25]. Furthermore, an increased urinary excretion of 3-MH in the catabolic state such as severe injury, thyrotoxicosis, cancer, and open-heart surgery were reported [13, 15, 26]. In this study, all patients had a normal diet and did not take glucocorticoids, but we could not control the potential confounders such as usual dietary protein level because we did not assess the daily dietary intake or dietary supplements. Also, a dietary protein restriction for this study might have affected the 3-MH/Cr.

In this study the CRP and IL-6 levels positively correlate with muscle proteolysis in the HF patients but not in controls. Our result is supported by a previous study which showed that IL-6 was as an independent predictor of an accumulated postoperative 3MH/Cr level in patients underwent open heart surgery [15]. Much evidence suggests that the pathogenesis of skeletal muscle loss in HF includes neurohormonal activation and inflammation [3, 4, 8]. A previous experimental study showed that the sympathetic nerve activation and increased catecholamine level in a mouse model of HF resulted in sarcomere loss, myocyte death, and capillary rarefaction [27]. Inflammatory cytokine signaling has been reported to exert skeletal myocyte injury, atrophy, and generation of reactive oxygen intermediates. Zhang et al. showed that rodents with a constant infusion of angiotensin II, which caused muscle loss with increasing IL-6 leads to insulin/insulin-like growth factor 1 (IGF-1) signaling suppression [28]. Dalla Libera et al. showed that treatment of IGF-1 improved the muscle atrophy and exercise capacity in a mouse model of HF [29]. Persistent elevations of inflammatory cytokines in sarcopenia patients were associated with impaired satellite cell regeneration [30] as well as reductions in endothelial reactivity and muscle perfusion, which are essential for muscle energetics and protein anabolism [31]. Finally, a recent report showed that CRP can contribute to an age-related decline in the muscle function by inhibiting muscle protein synthesis [32]. Our study also showed that the BNP level was associated with increased muscle proteolysis, suggesting a higher inflammatory status associated with severe HF may have led to excessive muscle proteolysis.

In this study, the random forest algorithm revealed that the BNP, LVEF, and inflammatory cytokines, in addition to the skeletal muscle-related parameters, played an important role in predicting the prognosis. These parameters had relatively greater effects on the prediction than the age, renal function, or hemoglobin levels, which have been hitherto considered important in predicting the prognosis. There have been many reports in which elevated levels of IL-6 or CRP have been observed in patients with HF and have a prognostic implication [33–35].

We showed that the random forest approach outperformed the logistic regression analysis and can be effectively applied to predictive modeling in HF despite the small number of observations and heterogeneous input parameters. Originally, the random forest approach was developed to explore the predictors of certain target phenomena by using a large amount of data and is now likely to become an alternate to explore predictors for the target phenomena in healthcare or the events in clinical medicine. The advantages of the random forest over the logistic regression are that it can deal with complicated combinations of different features and avoid overfitting. In addition, the random forest approach can provide a systematic and quantitative way to assess feature importance. The logistic regression, however, is difficult to deal with highly correlated covariates and assess the effect of each covariate. A logistic regression model is not able to be applied when there are more independent variables than observations. The random forest could overcome such difficulties. Our results using the logistic regression demonstrate that BNP, age, and systolic blood pressure were independent predictors. The random forest, however, shed light on the importance of the inflammatory cytokines and skeletal muscle wasting, which were undetected in the logistic regression. Although the importance of the skeletal muscle mass and strength in HF patients was suggested in our analysis, further research with other techniques is needed to understand the hierarchy in selected predictors and the link among the predictors.

We demonstrated that elderly HF patients, when compared with age- and sex-matched controls, had excessive skeletal muscle proteolysis and wasting and the possibility that inflammation is an important mechanism. The study showed that machine learning analytics using data from inflammation and skeletal muscle wasting can accurately predict outcomes. Optimizing the skeletal muscle mass and function in elderly HF patients has become an important issue to confer the quality of life and reduce the risk of death. Although effective treatments except exercise training currently remain to be established, future study is required to test whether pharmacological treatments that modulate the inflammatory signaling pathways that affect the muscle proteolysis and prognosis in elderly HF patients.

### Study limitations

This study was based on observations of a single-center, small cohort of patients in a university hospital. The small number of endpoints was a significant limitation of the prognostic study. Physical activity in daily life may vary between individuals and may consequently have confounded the inflammatory markers, skeletal muscle wasting, and functional capacity. The TNF-α is one of the major inflammatory cytokines in HF patients, but we did not measure the TNF-α level. For the inclusion criteria of the control subjects, we did not use the LVEF, but instead used a BNP < 100 pg/mL and no history of hospitalization due to worsening HF. This is because some of the elderly patients might have subclinical HF irrespective of LVEF at rest. Then, we considered that control elderly subjects should have no overt sign of HF or history of HF hospitalization at enrollment. However, those with an LVEF< 50% in the controls may develop HFrEF during long-term follow-up. We did not measure the fiber cross sectional area. The skeletal mass estimation using CT / MRI was more useful than ultrasonography, and ultrasonography may have limited their interpretation. The study patients were fully compliant with the dietary instructions, but some nutritional supplements may have affected the 3-MH. A recent study, however, suggested that meat intake does not affect the 3-MH [36].

## Conclusion

This study provided evidence of the association between inflammation and increased skeletal muscle proteolysis, reduced skeletal mass and strength, and their prognostic roles in elderly HF patients.

## Supplementary information

**Additional file 1 Supplementary file 1.** Covariates for the random forest analysis. **Supplementary file 2.** Logistic regression analysis, receiver operating characteristic curve, and Kaplan-Meier survival curve.

## Data Availability

The data analyzed during this study are available from the corresponding author on reasonable request.

## Abbreviations

CRP: High-sensitivity C-reactive protein
IL-6: Interleukin 6
BNP: B-type natriuretic peptide
3-MH: 3-methylhystidine
LVEF: Left ventricular ejection fraction
