# Supplementary material for: Association between inflammation and skeletal muscle proteolysis, skeletal mass and strength in elderly heart failure patients and their prognostic implications
Source: BMC Cardiovasc Disord. 2020 May 15;20:228. doi: 10.1186/s12872-020-01514-0 (PMC7229573; doi:10.1186/s12872-020-01514-0)
Supplement: Supplementary file 1 — Additional file 1 Supplementary file 1. Covariates for the random forest analysis. Supplementary file 2. Logistic regression analysis, receiver operating characteristic curve, and Kaplan-Meier survival curve. [file 12872_2020_1514_MOESM1_ESM.docx]

**Supplementary files**

Koshikawa M, et al. Association between inflammation and skeletal muscle impairment in elderly heart failure patients and their prognostic implications (Association of inflammation with skeletal muscle impairment in elderly heart failure patients and outcomes)

**Supplementary file 1.** Covariates for the random forest analysis.

**Supplementary file 2.** Logistic regression analysis, receiver operating characteristic curve, and Kaplan-Meier survival curve.

**Supplementary file 1.**

The following 29 baseline variables shown in Table 1 were used. Variables are followed by units, or categorical ranges.

• **Continuous covariates:**

Age (years)

BMI (kg/m2)

Systolic blood pressure (mmHg)

Diastolic blood pressure (mmHg)

Hemoglobin (g/dL)

Creatinine (mg/dL)

eGFR (mL/min/1.73m2)

CRP (mg/dL)

IL‐6 (pg/mL)

Creatine kinase (IU/L)

BNP (pg/mL)

LVEF (%)

LAD (mm)

• **Categorical covariates (all will be reduced to binary classification):**

Sex

Cardiac rhythm

Coronary artery disease

Valvular disease

Hypertension

Diabetes

Dyslipidemia

TIA/Stroke

β-Blocker

ACE-I/ARB

Loop diuretics

Aldosterone blocker

Calcium blocker

Statin

Aspirin

Anticoagulant

**Supplementary file 2.**

In the logistic regression variable selection, we employed a backward stepwise selection procedure in which the removal testing was based on the Akaike information criterion. We calculated the sensitivity, specificity, and area under the receiver operating characteristic (ROC) curve. The cut-off level for predicting the outcome was determined when it maximized the average of the sensitivity and specificity. The time-to-event curve describing the proportion of the patients remaining endpoint-free was calculated by the Kaplan-Meier method and compared with the log-rank test. The odds ratio and its 95% confidence interval (CI) are shown.

**Receiver operating characteristic curve (left panel) and Kaplan-Meier curve (right panel).**
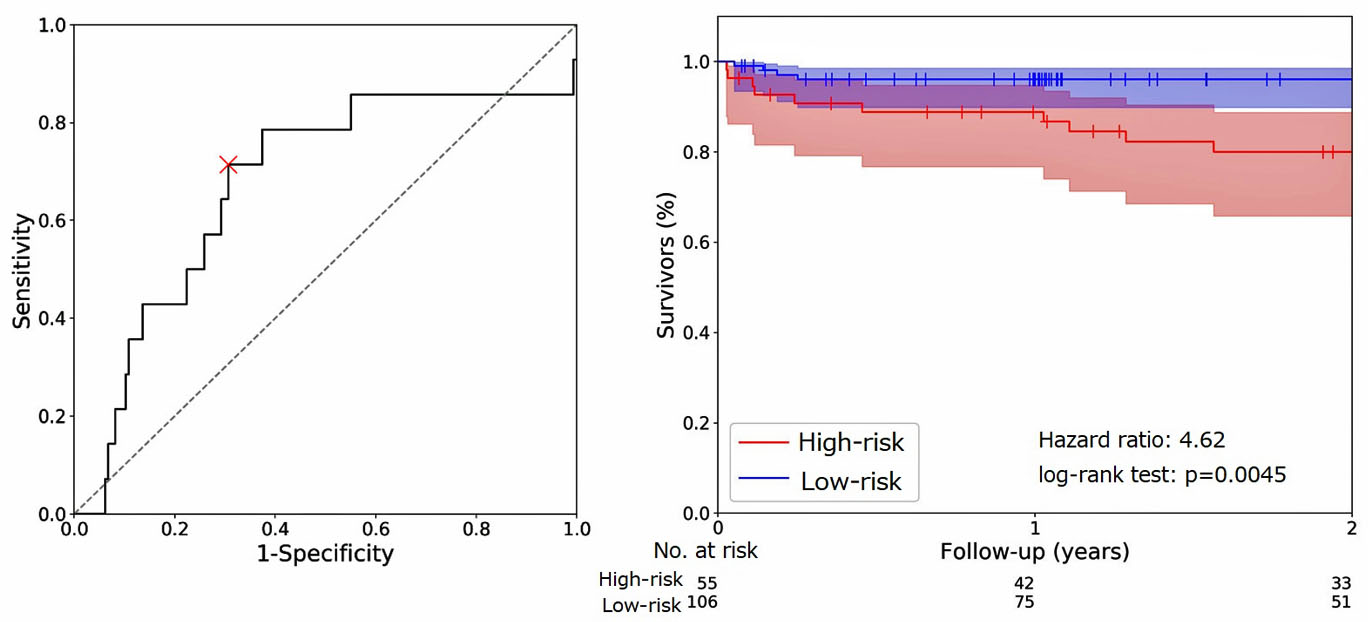


The cut-off value to dichotomize the patients in the high-risk group and low-risk group was 0.018. This had a sensitivity of 69.4% and specificity of 71.4%. The area under the ROC curve was 0.674 (95% CI 0.514 – 0.835), which was significantly lower than that in the random forest approach (p<0.01). The Kaplan-Meier analysis revealed that significantly higher event rate was observed in patients classified as a high-risk group.

**Table. Results of logistic regression analysis.**

| Variables | Odds ratio | 95% CI | P-value |
| --- | --- | --- | --- |
| BNP (pg/mL) | 5.17 | 4.53-5.89 | <0.05 |
| Age (years) | 1.11 | 1.10-1.12 | <0.05 |
| Systolic blood pressure (mmHg) | 0.927 | 0.923-0.932 | <0.01 |

Across the leave-one-out iterations of the logistic regression variable selection, the BNP, age and systolic blood pressure were statistically significant. The abbreviations are shown in Table 1.
